# Identifying disease associated genes by network propagation

**DOI:** 10.1186/1752-0509-8-S1-S6

**Published:** 2014-01-24

**Authors:** Yu Qian, Søren Besenbacher, Thomas Mailund, Mikkel Heide Schierup

**Affiliations:** 1 Bioinformatics Research Center, Aarhus University, 8000C Aarhus, Denmark

**Keywords:** Network analysis, Genome-wide association analysis, Network propagation

## Abstract

**Background:**

Genome-wide association studies have identified many individual genes associated with complex traits. However, pathway and network information have not been fully exploited in searches for genetic determinants, and including this information may increase our understanding of the underlying biology of common diseases.

**Results:**

In this study, we propose a framework to address this problem in a principled way, with the underlying hypothesis that complex disease operates through multiple connected genes. Associations inferred from GWAS are translated into prior scores for vertices in a protein-protein interaction network, and these scores are propagated through the network. Permutation is used to select genes that are guilty-by-association and thus consistently obtain high scores after network propagation. We apply the approach to data of Crohn's disease and call candidate genes that have been reported by other independent GWAS, but not in the analysed data set. A prediction model based on these candidate genes show good predictive power as measured by Area Under the Receiver Operating Curve (AUC) in 10 fold cross-validations.

**Conclusions:**

Our network propagation method applied to a genome-wide association study increases association findings over other approaches.

## Background

In recent years, genome-wide association studies (GWAS) have become a common tool to discover the genetic basis of complex diseases and have led to many scientific discoveries [1]. Many single nucleotide polymorphisms (SNPs) have been identified in a variety of diseases. The single marker analysis tests genetic association of individual SNPs and identifies only the most significant SNPs below a stringent significance level, for example, *p <*5 × 10^−8^, which is necessary to control the false positive rate on a genome-wide level. However, the identified SNPs only represent a small fraction of the genetic variants to contribute to complex diseases, due to small individual effect sizes. Markers that are truly but weakly associated with disease often fail to be detected [2].

It is well understood that the stability of biological systems is governed by many biomolecular interactions and multi-gene effects should be taken into consideration while mapping from genotypes to phenotypes. Consider a crucial biological mechanism, where failure of a small portion of the important genes can lead to dys-function of the whole biological mechanism. This is very likely to happen in case of complex disease, such as Crohn's Disease, and therefore multi-locus analysis show increase of power when analyzing such data.

Pathway-based or gene set enrichment analysis has become a potentially powerful approach in search of disease associated genes (for a recent review of pathway analysis, see [3]). One of the most popular methods is GSEA [4]. Using a modified Kolmogorov-Smirnov test, it compares the *p *value distribution of genes in a pathway with the rest of the genes. GSEA has successfully identified the IL-12/IL-23 pathway that is significantly associated to Crohn's disease [5]. However, there are some disadvantages to the common pathway based analysis. First, most of the studies choose the most significant SNP from each gene as a representative, and therefore systematic but small changes in a gene-set will be missed if individual genes do not have any SNPs with strong marginal association. Second, how robust the methods are with regard to factors such as pathway annotations and pathway size is not clear. Third, many methods treat all genes equally despite that some genes (e.g., housekeeping genes) appear in many pathways [6]. The additional information of genes in overlapping pathways should not be ignored, as shown in a study where weighting genes based on their appearance in the gene sets can improve gene set ranking and boost sensitivity of the analysis [7].

Similar to pathway-based analysis, where biologically relevant connections from public databases are utilised in GWAS, network-based analysis has also become a popular tool for the study of complex disease. In context of molecular interaction networks, it has been found that about one third of known disorders with multiple genes show physical clustering of genes with the same phenotype and these clusters are likely to represent disorder-specific functional modules [8]. A concept of disease modules was emerged as more studies show that proteins that are involved in the same disease show a high propensity to interact with each other [9,10]. If a few disease components are identified, other disease-related components are likely to be found in their network-based vicinity. For example, various module search methods have been developed in search of disease associated modules [11,12]. However, a module consists of an arbitrary number of genes, it often requires intensive simulations for multiple-testing corrections.

Network Guilt by Association (GBA) is an approach for identifying disease genes based on the observation that similar phenotypes arise from functionally related genes. Algorithms related to Google's PageRank, such as Iterative Ranking and Gaussian smoothing, are applied in prioritizing candidate disease genes using network information [13]. A typical workflow looks like this: given a query disease, known causal genes of diseases that are phenotypically similar to the query disease are given a prior score in human PPI network, then the prior scores are propagated and smoothed over network such that each protein gets an association score. Genes with high association scores are considered as candidate associations.

In this study, we analyzed a GWAS in a GBA framework. The network is overlaid with GWAS information, that is, each gene is assigned a prior score based on the gene level *p *value from GWAS, which represents our prior knowledge of its association to the disease. After propagation, the prior score has been smoothed over the whole network and each gene gets a new association score, denoted as the posterior score, with higher posterior score representing stronger evidence for association. If a gene has many neighbours that are associated with the disease, it is very likely that itself is also associated. A gene with high posterior score can be called as candidate genes, even though it has a low prior score and fails to be called in standard GWAS due to stringent *p *value cut-off, chip coverage or sampling bias.

A recent study applied similar ideas to prioritize candidate disease genes and demonstrated a boost in the power to detect associated genes in GWAS [14]. Using a naive Bayes framework on datasets of Crohn's disease and Type 1 diabetes, the posterior score of each gene was obtained by adding its own log odds from GWAS (as prior) and a soft GBA score from neighbors. The study showed that some genes with high posterior scores are actually validated as true associations in later studies, although they do not have highest prior scores (e.g., lowest *p *values in GWAS studies) and would possibly be ignored in two-stage studies. However, there are some open questions in this study. First, the posterior score of a gene depends not only on its neighbors association of disease, but also on how many neighbors it has, i.e, network topology. It is appealing to mark a gene with high posterior score as associated, neglecting the fact that a high posterior score is merely due to a high degree (receiving information from more neighbors). Second, there is no statistic control (e.g., false discovery rate) for the findings. If there is no signal in the network, i.e, a completely random prior for all the genes, this method still outputs the genes with highest posterior scores.

## Methods

In this section, we first describe the data we used and the network propagation framework, then we build a prediction model based on the GBA genes, and evaluate the performance in cross-validation (CV).

### Data Set

**Prior information from GWAS**. We analyzed the raw anonymous genotype data of the Wellcome Trust Case Control Consortium (WTCCC) study. The original cohort includes 2005 Caucasian UK patients of Crohn's disease and 3004 controls genotyped on the Affymetrix 500K mapping array. The details are described in [15]. Genotypes with posterior probability (or CHIAMO score) lower than 0.98 are considered as missing data. Markers are removed if the percentage of missing data was larger than 5% or if they are not in Hardy-Weinberg equilibrium (*p *> 0.0001 for control group). We further remove some individuals with missing allele larger than 3% or of non-European ancestry or with duplicated samples, as suggested by [15], and are left with 1748 cases and 2938 controls. Finally we map a SNP to a gene if it was located within the gene or 10kb immediately upstream or downstream.

**Interaction network**. The PPI network is built based on the STRING database version 9.0 [16]. Only interactions with a score larger than 700 are included, and it results in 229599 interactions involving 15010 proteins. We use proteins and genes interchangeably in the following, because SNPs were first mapped to genes then mapped to corresponding proteins. The GWAS dataset only covers part of the genes in the network, for example, 11363 genes for Crohn's disease. We discard all the vertices that are not covered by GWAS, keeping only edges between covered vertices. Isolated nodes are also removed, in the end, we are left with a large connected network *N *.

### Propagation of evidence

Consider a PPI network as an undirected graph *G *= (*V*, *E*, *w*), where nodes *V *are a set of proteins, edges *E *are links between proteins if interaction exists, *w *is the weight of an edge. For a node *v *∈ *V*, denote its total number of neighbors by *degree*(*v*) and its direct neighbors in *G *by *N *(*v*). Let $Y:V\to {\mathbb{R}}_{\geq 0}$ represent a function of prior evidence, i.e., assign high score to a node *v *if we a priori (from GWAS) believe that it is associated to the disease. $F:V\to {\mathbb{R}}_{\geq 0}$ denotes a function of posterior evidence, i.e., *F*(*v*) represents the posterior evidence of association after propagating the information of its neighbor nodes. The main three steps of network propagating are, (1) obtaining prior information from GWAS, (2) calculating and normalising posterior scores by network propagation (with choice of tuning parameters), (3) selecting genes with highest posterior score as candidates.

**Gene-level prior scores**. The *p *value of a gene is defined as the minimum single marker test *p *value of its SNPs, as widely used in pathway analysis. The prior score of gene *i *defined as *y_i _*= Φ^−1 ^(1 − *p_i_/*2) and Φ is the Cumulative Distribution Function of normal distribution. Therefore, under the null hypothesis of no association, $y_{i}\sim N^{+}\left(0,1\right)$. According to [17], minimum *p *values performs best in most scenarios, we also tried Fisher's combined probability test, however, it gives lower internal consistency of gene ranks for random subset of the data.

**Calculating posterior scores by propagation**. The posterior score *F *is computed as

$$F\left(v\right)=\alpha \left[\underset{u\in N\left(v\right)}{\sum }F\left(u\right){w^{\prime }}_{v,u}\right]+\left(1-\alpha \right)Y\left(v\right)$$

where the parameter *α*∈ (0, 1) weights the relative importance of information received from neighbors, and $w_{v,u}^{\prime }=w_{v,u}/\sqrt{d\left(v\right)\times d\left(u\right)}$ denotes the weight of edges, with *d*(*v*) the degree of node *v*. The above formula can be expressed in linear form *F *= *αW*'*F *+ (1 − *α*)*Y*, which is equivalent to

(1) $$F={\left(I-\alpha W^{\prime }\right)}^{-1}\left(1-\alpha \right)Y$$

It can be proved that *W*' is similar to a stochastic matrix, which has eigenvalues in [−1, 1] (according to the PerronFrobenius theorem). Since *α*∈ (0, 1), the eigenvalues of (*I *− *αW*')^−1^ exists. Though the linear equation can be solved analytically, it is difficult to compute the inverse of a large matrix with |*V*| × |*V*| dimension, and we choose a iterative propagation method to solve the system. At iteration *t*, we compute

(2) $$F^{t}=\alpha W^{\prime }F^{t-1}+\left(1-\alpha \right)Y$$

**Tuning parameters**. There are two tuning parameters in the model, *α*and *T*. *T *denotes the number of iterations of propagation. We study two extreme scenarios, *T *= 1 and *T *= ∞. When *T *= 1, each node only receives information from its direct neighbors. Posterior score *F *is calculated from equation (2). When *T *= ∞, equation (2) reaches equilibrium after many iterations and the information is smoothed over the network, therefore each node also gains information from its indirect neighbors through iterations. *F *scores will converge as shown in equation (1). In practice, equilibrium is often achieved within 20 iterations.

*α*, also known as the damping parameter in the literature, denotes how much information a node receives from neighbors. Higher *α*indicates less weight on its own prior information. Previous applications of similar algorithms of ranking SNPs or genes recommend *α*∈ [0.5, 0, 95] [13,18]. We explored *α*∈ (0.2, 0.4, 0.6, 0.8, 0.9) in the experiments. A good choice of *α*should give better internal consistency of gene rank by posterior score, which can be measured by Kendall Tau rank correlation [19].

We randomly choose half of the case and control samples and rank genes based on posterior scores. Low consistency is obtained for *α *∈ (0.2, 0.4) and it agrees with a previous study that *α *should be more than 0.5 [18]. Highest consistency is obtained when *α*∈ (0.8, 0.9), thus we choose the mean *α*= 0.85 in the main analysis, unless otherwise specified.

### Identifying associated genes

As shown in Equation (2), a gene can have a high posterior score under two conditions: (1) it has a high degree in the network and receives more information from its neighbors than the other low degree genes, (2) most of its neighbors are associated with the disease and itself is GBA. Therefore ranking a gene based on posterior score has some issues when the first condition is dominant, we may include too many false positives in the candidate gene set and have a potential power loss for genes with lower degrees in the network.

Here we suggest a framework, that uses permutations to identify GBA genes and eliminates potential false positives. The pseudocode is shown in the following. Input parameters include: significant threshold and number of permutations, *K*.

example input: Sig. Thresh.=0.01, *K*=10000;

// GWAS prior

prior scores based on *p *values of Cochran-Armitage trend test in GWAS data;

propagate prior scores by equation (2);

normalize posterior scores for all genes so that the sum is 1;

record posterior score for gene*_i _*as $S_{0}^{i}$;

// permutation

for *k *in 1 to *K*; do

permute case and control labels, calculate prior scores from *p *values;

propagate prior scores by equation (2);

normalize posterior scores for all genes so that the sum is 1;

record posterior score for gene*_i _*as $S_{k}^{i}$;

done

// find candidate GBA genes

for gene*_i _*in network; do

the *p *value of the posterior score of gene*_i _*is $\frac{\sum_{k=1}^{K}I\left(S_{k}^{i}>S_{0}^{i}\right)}{K}$

gene*_i _*is candidate association if the *p *value of its posterior score is smaller than Sig. Thresh.

done

### Prediction model and ROC

The GBA candidate genes are important nodes in the network, because many of their neighboring genes are associated with disease. To measure how the GBA genes collectively contribute to the disease, we used them to build prediction models and evaluate the performance in 10-fold cross-validation (CV). The prediction models based on 90% of the cases and controls were tested on the remaining 10% data, and it was repeated 10 times with different 90% and 10% of the cohorts.

A logistic regression model with all the SNPs as covariates is fitted by the R package *glmnet *[20]. Though the GBA framework only chose the most significant SNP to represent the gene, we used all the SNPs located within 10Kb boundary of the candidate genes. *glmnet *applies cyclical coordinate descent to solve elastic-net penalized regression models, which are mixtures of two penalties: *l*_1 _(the lasso) and *l*_2 _(ridge regression), and it generates models with relatively few predictors. To evaluate the performance of the predictive model, we calculated the average Area Under the receiver operating characteristic Curves (AUC) [21] for all 10 trials.

## Results and discussion

### Problems with association by rank

Network propagation for prioritizing associated genes has been applied in several studies when there is functional similarity between a given gene and the known disease gene. The selected few known disease associated genes give prior information for the network, after propagation, each gene gets a posterior score, which represents its association to the disease.

Implementation of similar ideas in a GWAS showed boosting in identification of disease-associated genes [14]. However, in such an application, genes of high degree often have high posterior scores due to propagation. If we simply take the genes with highest posterior score as candidates, we may end up including too many false positives in the candidate gene list. In Additional file 1: Table S1, where *N*_0 _refers to network with the GWAS prior and *N*_*k*≠0 _to networks with randomized prior, one can see that the top ranked genes in *N*_0 _are also often ranked top in *N*_*k*≠0_. Although the detailed implementation of our study and the one by [14] is different, it reveals the necessity of utilizing such network methods in a more cautious way. Moreover, most of the top ranked genes in *N*_0 _have high network degree, with average degree of 195.4, while the PPI used in our studies has an average degree of 24.5, it again confirms our concern that genes of high degree tend to be ranked on top in the network.

### Candidate genes

#### Identified candidate genes

**Figure 1:**
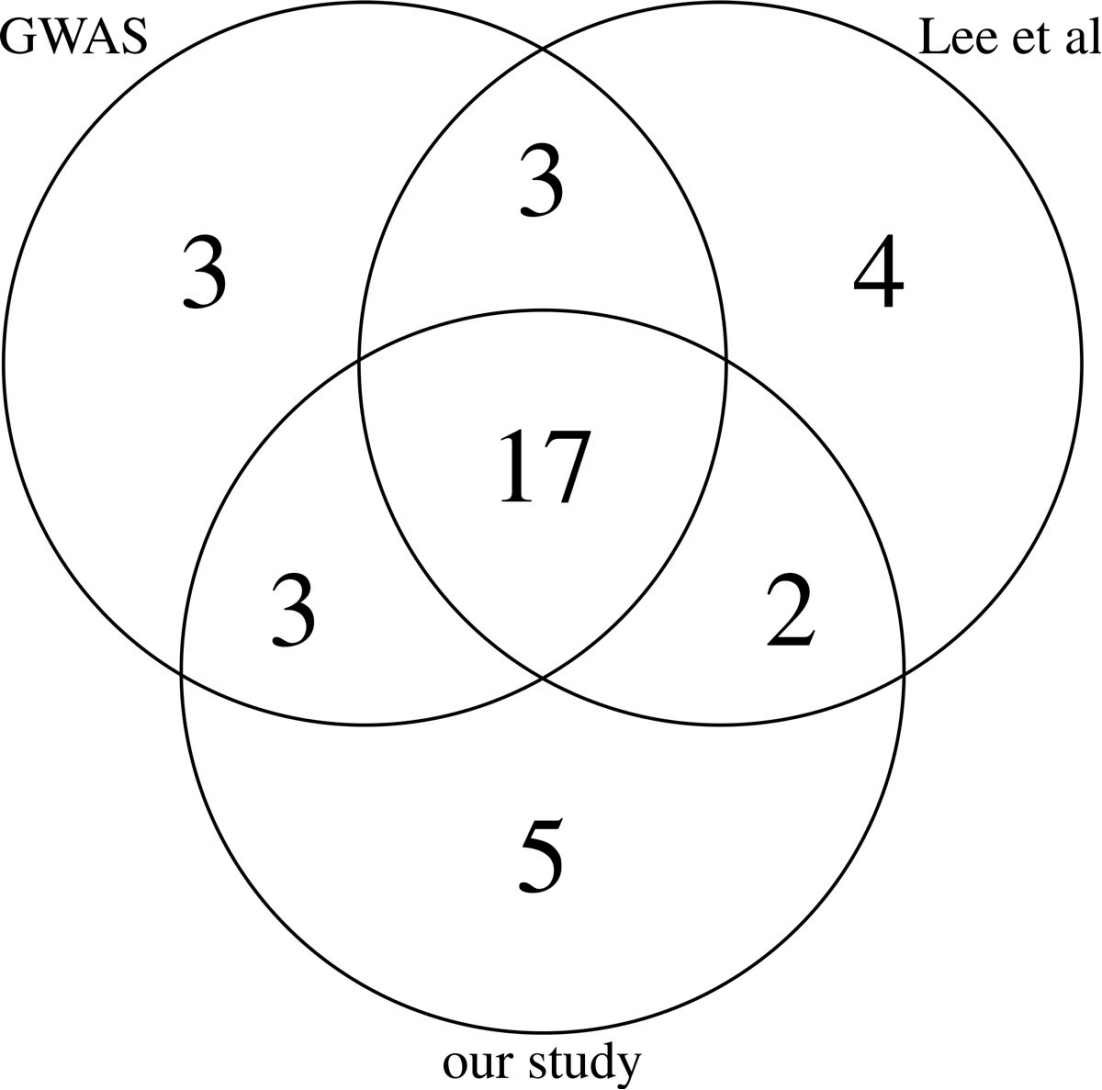
**Number of reported genes**. Overlap of validated genes among top 150 genes for each method, all of them map SNPs to genes within 10kb boundary. Validated genes denote genes that are reported as candidate in later GWAS studies. Method GWAS means ranking genes by minimum *p *value.

The study by [14] listed the top-ranked 150 genes, to make the results comparable, we also made a list of 150 top-ranked genes with *T *= 1, they are ranked by the *p *value of their posterior score obtained from permutation. As shown in Additional file 1: Table S2, one can see that many genes with significant *p *value from GWAS are also called in our study. This is not surprising since the prior scores from GWAS contribute to the posterior scores. Moreover, our method also identified some genes that are not called by standard methods in this data set, but reported in other independent GWAS. The number of genes that are validated by other studies and reported in GWAS catalog [22] is shown in Figure 1. There are 7 genes identified as association candidates in our study, which failed to be called by GWAS *p *value ranking, they are PTPN22, IRF1, PTGER4, IL12B, IL18R1, FASLG and JAK2. Except IL12B and JAK2, the other 5 genes also failed to be called by [14]. These candidate genes all have a higher rank of posterior scores in the network of GWAS prior, compared to network with random prior, as shown in Additional file 1: Table S2. For example, gene JAK2 (MIM 147796) has a gene level *p *value of 0.0523, but its rank in the network increases from average of 104 in *N*_*k*≠0 _to 54 in *N*_0_. IL18R1 (MIM 604494) has a GWAS *p *value of 0.003, and its rank of posterior score is 3342 in *N*_*k*≠0 _and 1109 in *N*_0_. This gene would be missed by both single marker test as well as ranking genes by posterior score.

There are 4 genes, C13orf31, MST1, RTEL1 and HLA-DQA2, that were listed on top 150 by [14] and failed to be called in our study. The reason we failed to call them is because these genes are not present in the STRING database we used. Therefore a more complete PPI database integrated from diverse sources is needed, as was done by [14].

Based on the number of significant genes that are reported by other independent GWAS or meta-analyses, there seems to be no big advantages of our results compared with the one by [14]. However, these studies are designed to discover different signals in the network. Our method focus more on genes with abundant guilty neighbors, thus it has higher power to discover local signals even with low degree genes but less power to call high degree genes. However, there are also two other factors in the approaches that may contribute to the difference in performance. First, the detailed implementation is different, [14] rank genes by posterior log odds, and choose a best prior log odds such that the number of validated genes is maximized. Second, the reported genes in GWAS catalog might have a bias to genes that have significant *p *value in certain type of study (eg., certain type of chips). Nevertheless, our study showed potential power of GBA framework to boost GWAS signals.

#### Genomic prediction model

We further investigated whether the candidate genes collectively contribute to the disease and evaluated the extent to which predictions were driven by these candidate genes. A previous study conducted pathway analysis on the same data set [23] and built a logistic regression model of 277 genes with a variable selection algorithm. The average AUC was 0.6 in 10-fold CV with all the SNPs within 10kb boundary of the selected genes, and it dropped to 0.56 after excluding all SNPs with *p *value *<*5 × 10^−7^. Using GBA candidate genes, we had higher average AUC in 10-fold CV, the average is 0.705 (T = 1) and 0.730 (T=*∞*) in models including all the SNPs that are mapped to candidate genes, and 0.687 (T = 1) and 0.715 (T=*∞*) after removing SNPs with *p *value *<*5 × 10^−7^. The numbers are shown in Table 1, one can see that the increased AUC is not due to the number of genes we used to build the model, on the contrary, we used fewer genes for the prediction model, with 130 (T = 1) and 184 (T=*∞*) genes respectively.

**Table 1:** Prediction models based on candidate genes.

	T = 1					T=*∞*				
method	Gene*^a^*	SNP1*^b^*	SNP2*^c^*	AUC1*^d^*	AUC2*^e^*	Gene	SNP1	SNP2	AUC1	AUC2
Sig. Thresh.*^f^*	130	1176	1145	0.705(0.033)	0.687(0.032)	184	1314	1281	0.730(0.017)	0.715(0.013)
PS*^g^*	130	2331	2310	0.645(0.024)	0.627(0.026)	184	3279	3256	0.645(0.024)	0.626(0.026)

*^a^*Gene is number of genes identified as GBA associations,

*^b^*SNP1 is number of SNPs mapped to these genes.

*^c^*SNP2 is *^b^*SNP1 excluding snps that reach GWAS significance of 5 × 10^−7^.

*^d^*AUC1, mean AUC of prediction models built with *^b^*SNP1, numbers in parentheses are standard deviation in cross validations.

*^e^*AUC2, similar to AUC1, prediction models built with *^c^*SNP2.

*^f^*Sig. Thresh. = 0.01

*^g^*PS means genes with highest posterior scores are selected.

We also built prediction models with the same number of genes that are ranked on top (based on posterior scores), which would be called candidate genes in the study of [14]. As shown in Table 1, there is a AUC drop with models of top ranked genes. The reason might be that the candidate genes in our study are GBA genes and collectively contribute to disease, while top ranked genes can be special in the network topology but with no association to the disease.

### Boosting signal from IL12 pathway genes

Many studies of pathway analysis have uncovered significant associations between Crohn's disease and the IL12/23 signaling pathway [5,24]. 19 of 20 genes in IL12 pathway are included in our network analysis, most of them have a posterior score (rank) increase in *N*_0_, shown in Additional file 1: Table S3. As propagation redistributes the information of the network, standard pathway enrichment analysis based on posterior scores might have an advantage over the one based on direct GWAS results.

## Conclusions

Combining GWAS data with function databases is very appealing as it provides more explanatory power for the list of candidate genes. While pathway methods have shown success in many applications, they also have limitations. For example, genes involved in multiple pathways might introduce bias in different pathways, different definitions of the same pathway in different knowledge bases can affect performance assessment in terms of power and true positive/negative rate. [3].

The methods of combining GWAS and PPI networks mainly fall into two categories, (1) dense module search algorithms in search of significantly enriched subnetworks [11,12,25], (2) propagation algorithms related to Google's PageRank [13,14] in search of genes that have top ranks in the network. However, while methods in group (1) require intensive randomization of network topology for accessing module significance, and often encounter multiple testing problem in searching of modules of various sizes in high dimension space, methods in group (2) fail to distinguish signals from GWAS and signals from network, and therefore tend to have a high false positive rate, especially in case of biased PPI database.

Our study extends the idea of network propagation with GWAS information, such that information from various resources can be utilized. The performance of this method can be improved in various ways. Integration of diverse data sources, as suggested in [14], will improve the ability to prioritize disease genes. Mapping multiple SNPs to a single gene is the simplest way of obtaining genelevel statistics, yet some collapsing-based and kernelbased methods are worth trying for gene-level statistics [26]. There are also potential extensions of this study. For example, most of the 20 major genes in IL12 pathway, identified as associated in Crohn's Disease, have increased posterior score in the network of GWAS prior, it implies that network propagation method redistributes the information in the network where the true associations get enriched information. Therefore pathway analysis based on posterior scores may have more power than the standard pathway analysis. Many methods that detect interaction and epistasis, such as Support Vector Machine [27] and Logic Regression [28] are not applicable in genome-wide scale due to high dimensions, a reduced search space such as interactions among GBA genes might yield some results.

## Competing interests

The authors declare that they have no competing interests.

## Authors' contributions

The project was conceived by YQ, SB, TM and MS. SB prepared the original data, YQ designed and conducted the analysis. YQ drafted the manuscript, SB, TM and MS shared in writing the manuscript. All authors have read and approved the final manuscript.

## Supplementary Material

Additional file 1 **Genes with highest posterior scores**. Table S1 lists genes with highest posterior scores in the network, with parameter of *T *= 1. Candidate gene set of top 150 genes. Table S2 lists the top 150 candidate GBA genes in our study, which are used for comparison with other methods. Genes in IL12 pathway. Table S3 lists 19 genes in IL12 pathway, most of them have an increased posterior score in the network of GWAS prior. Click here for file

## Declarations

Publication of this article was funded by EU Marie Curie action (NextGene).

This article has been published as part of *BMC Systems Biology *Volume 8 Supplement 1, 2014: Selected articles from the Twelfth Asia Pacific Bioinformatics Conference (APBC 2014): Systems Biology. The full contents of the supplement are available online at http://www.biomedcentral.com/bmcsystbiol/supplements/8/S1.

## References

[B1] Visscher PM Brown MA McCarthy MI Yang J Five Years of GWAS Discovery The American Journal of Human Genetics 2012 90 7 24 http://linkinghub.elsevier.com/retrieve/pii/S0002929711005337 10.1016/j.ajhg.2011.11.029 PMC3257326 22243964

[B2] Jia P Wang L Meltzer HY Zhao Z Pathway-based analysis of GWAS datasets: effective but caution required The International Journal of Neuropsychopharmacology 2010 14 04 567 572 http://www.journals.cambridge.org/abstract\_S1461145710001446 21208483 10.1017/S1461145710001446

[B3] Khatri P Sirota M Butte AJ Ten Years of Pathway Analysis: Current Approaches and Outstanding Challenges PLoS Computational Biology 2012 8 2 e1002375 http://dx.plos.org/10.1371/journal.pcbi.1002375 10.1371/journal.pcbi.1002375 22383865 PMC3285573

[B4] Subramanian A Tamayo P Mootha VK Mukherjee S Ebert BL Gillette Ma Paulovich A Pomeroy SL Golub TR Lander ES Mesirov JP Gene set enrichment analysis: a knowledge-based approach for interpreting genome-wide expression profiles Proceedings of the National Academy of Sciences of the United States of America 2005 102 43 15545 15550 http://www.pubmedcentral.nih.gov/articlerender.fcgi?artid=1239896&tool=pmcentrez&rendertype=abstract 10.1073/pnas.0506580102 16199517 PMC1239896

[B5] Wang K Zhang H Kugathasan S Annese V Bradfield JP Russell RK Sleiman PMa Imielinski M Glessner J Hou C Wilson DC Walters T Kim C Frackelton EC Lionetti P Barabino A Van Limbergen J Guthery S Denson L Piccoli D Li M Dubinsky M Silverberg M Griffiths A Grant SFa Satsangi J Baldassano R Hakonarson H Diverse genome-wide association studies associate the IL12/IL23 pathway with Crohn Disease American journal of human genetics 2009 84 3 399 405 http://www.pubmedcentral.nih.gov/articlerender.fcgi?artid=668006&tool=pmcentrez&rendertype=abstract 10.1016/j.ajhg.2009.01.026 19249008 PMC2668006

[B6] Ma J Sartor Ma Jagadish HV Appearance frequency modulated gene set enrichment testing BMC bioinformatics 2011 12 81 http://www.ncbi.nlm.nih.gov/pubmed/21418606 10.1186/1471-2105-12-81 21418606 PMC3213687

[B7] Tarca AL Draghici S Bhatti G Romero R Downweighting overlapping genes improves gene set analysis BMC bioinformatics 2012 13 136 http://www.pubmedcentral.nih.gov/articlerender.fcgi?artid=3443069&tool=pmcentrez&rendertype=abstract 10.1186/1471-2105-13-136 22713124 PMC3443069

[B8] Feldman I Rzhetsky A Vitkup D Network properties of genes harboring inherited disease mutations Proceedings of the National Academy of Sciences of the United States of America 2008 105 11 4323 4328 http://www.pubmedcentral.nih.gov/articlerender.fcgi?artid=2393821&tool=pmcentrez&rendertype=abstract 10.1073/pnas.0701722105 18326631 PMC2393821

[B9] Barabási AL Gulbahce N Loscalzo J Network medicine: a network-based approach to human disease Nature reviews Genetics 2011 12 56 68 http://www.ncbi.nlm.nih.gov/pubmed/21164525 10.1038/nrg2918 21164525 PMC3140052

[B10] Cai JJ Borenstein E Petrov Da Broker genes in human disease Genome biology and evolution 2010 2 815 25 http://www.pubmedcentral.nih.gov/articlerender.fcgi?artid=2988523&tool=pmcentrez&rendertype=abstract 10.1093/gbe/evq064 20937604 PMC2988523

[B11] Jia P Wang L Fanous AH Pato CN Edwards TL Zhao Z Network-Assisted Investigation of Combined Causal Signals from Genome-Wide Association Studies in Schizophrenia PLoS computational biology 2012 8 7 e1002587 http://www.ncbi.nlm.nih.gov/pubmed/22792057 10.1371/journal.pcbi.1002587 22792057 PMC3390381

[B12] Akula N Baranova A Seto D Solka J Nalls Ma Singleton A Ferrucci L Tanaka T Bandinelli S Cho YS Kim YJ Lee JY Han BG McMahon FJ A network-based approach to prioritize results from genome-wide association studies PloS one 2011 6 9 e24220 http://www.pubmedcentral.nih.gov/articlerender.fcgi?artid=3168369&tool=pmcentrez&rendertype=abstract 10.1371/journal.pone.0024220 21915301 PMC3168369

[B13] Vanunu O Magger O Ruppin E Shlomi T Sharan R Associating genes and protein complexes with disease via network propagationplos computational biology 2010 6 e1000641 http://www.pubmedcentral.nih.gov/articlerender.fcgi?artid=2797085&tool=pmcentrez&rendertype=abstract 10.1371/journal.pcbi.1000641 20090828 PMC2797085

[B14] Lee I Blom UM Wang PI Shim JE Marcotte EM Prioritizing candidate disease genes by networkbased boosting of genome-wide association data Genome research 2011 21 7 1109 21 http://www.pubmedcentral.nih.gov/articlerender.fcgi?artid=3129253&tool=pmcentrez&rendertype=abstract 10.1101/gr.118992.110 21536720 PMC3129253

[B15] WTCCC Genome-wide association study of 14,000 cases of seven common diseases and 3,000 shared controls Nature 2007 447 7145 661 78 http://www.ncbi.nlm.nih.gov/pubmed/17554300 10.1038/nature05911 17554300 PMC2719288

[B16] Szklarczyk D Franceschini A Kuhn M Simonovic M Roth A Minguez P Doerks T Stark M Muller J Bork P Jensen LJ von Mering C The STRING database in 2011: functional interaction networks of proteins, globally integrated and scored Nucleic acids research 2011 39 Database D561 8 http://www.pubmedcentral.nih.gov/articlerender.fcgi?artid=3013807&tool=pmcentrez&rendertype=abstract 10.1093/nar/gkq973 21045058 PMC3013807

[B17] Chapman J Whittaker J Analysis of multiple SNPs in a candidate gene or region Genetic Epidemiology 2009 32 6 560 566 18428428 10.1002/gepi.20330 PMC2691454

[B18] Davis Na Crowe JE Pajewski NM McKinney Ba Surfing a genetic association interaction network to identify modulators of antibody response to smallpox vaccine Genes and immunity 2010 11 8 630 636 http://www.pubmedcentral.nih.gov/articlerender.fcgi?artid=3001955&tool=pmcentrez&rendertype=abstract 10.1038/gene.2010.37 20613780 PMC3001955

[B19] McKinney Ba Pajewski NM Six Degrees of Epistasis: Statistical Network Models for GWAS Frontiers in genetics 2011 2 January 109 http://www.pubmedcentral.nih.gov/articlerender.fcgi?artid=3261632&tool=pmcentrez&rendertype=abstract 22303403 10.3389/fgene.2011.00109 PMC3261632

[B20] Friedman J Hastie T Tibshirani R Regularization Paths for Generalized Linear Models via Coordinate Descent Journal of Statistical Software 2010 33 PMC2929880 20808728

[B21] Graham NE Areas beneath the relative operating characteristics (ROC) and relative operating levels (ROL) curves: Statistical signi cance and interpretation Quarterly Journal of the Royal Meteorological Society 2002 2145 2166

[B22] Hindorff La Sethupathy P Junkins Ha Ramos EM Mehta JP Collins FS Manolio Ta Potential etiologic and functional implications of genome-wide association loci for human diseases and traits Proceedings of the National Academy of Sciences of the United States of America 2009 106 23 9362 7 http://www.pubmedcentral.nih.gov/articlerender.fcgi?artid=2687147&tool=pmcentrez&rendertype=abstract 10.1073/pnas.0903103106 19474294 PMC2687147

[B23] Eleftherohorinou H Wright V Hoggart C Hartikainen AL Jarvelin MR Balding D Coin L Levin M Pathway analysis of GWAS provides new insights into genetic susceptibility to 3 inflammatory diseases PloS one 2009 4 11 e8068 http://www.pubmedcentral.nih.gov/articlerender.fcgi?artid=2778995&tool=pmcentrez&rendertype=abstract 10.1371/journal.pone.0008068 19956648 PMC2778995

[B24] Benson JM Sachs CW Treacy G Zhou H Pendley CE Brodmerkel CM Shankar G Mascelli Ma Therapeutic targeting of the IL-12/23 pathways: generation and characterization of ustekinumab Nature biotechnology 2011 29 7 615 24 http://www.ncbi.nlm.nih.gov/pubmed/21747388 10.1038/nbt.1903 21747388

[B25] Jia P Zheng S Long J Zheng W Zhao Z dmGWAS: dense module searching for genome-wide association studies in protein-protein interaction networks Bioinformatics 2011 27 Oxford, England 95 102 http://www.pubmedcentral.nih.gov/articlerender.fcgi?artid=3008643&tool=pmcentrez&rendertype=abstract 10.1093/bioinformatics/btq615 21045073 PMC3008643

[B26] Li L Zheng W Lee JS Zhang X Ferguson J Yan X Zhao H Collapsing-based and kernel-based single-gene analyses applied to Genetic Analysis Workshop 17 mini-exome data BMC proceedings 2011 5 Suppl 9 Suppl 9 S117 http://www.pubmedcentral.nih.gov/articlerender.fcgi?artid=3287841&tool=pmcentrez&rendertype=abstract 22373309 10.1186/1753-6561-5-S9-S117 PMC3287841

[B27] Ban HJ Heo JY Oh KS Park KJ Identification of type 2 diabetes-associated combination of SNPs using support vector machine BMC genetics 2010 11 26 http://www.pubmedcentral.nih.gov/articlerender.fcgi?artid=2875201&tool=pmcentrez&rendertype=abstract 10.1186/1471-2156-11-26 20416077 PMC2875201

[B28] Kooperberg C Ruczinski I LeBlanc ML Hsu L Sequence analysis using logic regression Genetic epidemiology 2001 21 Suppl 1 Suppl 1 S626 31 http://www.ncbi.nlm.nih.gov/pubmed/11793751 11793751 10.1002/gepi.2001.21.s1.s626

