# Supplementary material for: Identifying disease associated genes by network propagation
Source: BMC Syst Biol. 2014 Jan 24;8(Suppl 1):S6. doi: 10.1186/1752-0509-8-S1-S6 (PMC4080512; doi:10.1186/1752-0509-8-S1-S6)
Supplement: Additional file 1 — Genes with highest posterior scores. Table S1 lists genes with highest posterior scores in the network, with parameter of T = 1. Candidate gene set of top 150 genes. Table S2 lists the top 150 candidate GBA genes in our study, which are used for comparison with other methods. Genes in IL12 pathway. Table S3 lists 19 genes in IL12 pathway, most of them have an increased posterior score in the network of GWAS prior. [file 1752-0509-8-S1-S6-S1.pdf]

| name | [a]rank_N0 | [b]rank_random | [c]degree | [d]validate |
|---|---|---|---|---|
| TP53 | 0 | 0.0 | 615 | N |
| CDC42 | 1 | 3.4 | 258 | N |
| FAM48A | 2 | 1.1 | 521 | N |
| INS-IGF2 | 3 | 3.0 | 461 | N |
| AKT1 | 4 | 4.9 | 497 | N |
| PLEK | 5 | 4.2 | 140 | N |
| RAC1 | 6 | 6.3 | 290 | N |
| CALM1 | 7 | 6.9 | 265 | N |
| EGFR | 8 | 9.4 | 344 | N |
| ESR1 | 9 | 10.9 | 287 | N |
| EGF | 10 | 10.4 | 374 | N |
| DNAH8 | 11 | 14.9 | 148 | N |
| CTNNB1 | 12 | 7.4 | 369 | N |
| IL6 | 13 | 16.2 | 397 | N |
| NFKB1 | 14 | 15.0 | 400 | N |
| STAT3 | 15 | 46.5 | 282 | Y |
| JUN | 16 | 18.8 | 394 | N |
| SP1 | 17 | 12.4 | 352 | N |
| DLG4 | 18 | 17.7 | 70 | N |
| IL2 | 19 | 24.3 | 309 | N |
| FOS | 20 | 31.5 | 342 | N |
| MYC | 21 | 17.7 | 370 | N |
| FYN | 22 | 19.5 | 233 | N |
| EP300 | 23 | 27.0 | 260 | N |
| GRB2 | 24 | 41.8 | 242 | N |
| APP | 25 | 32.8 | 334 | N |
| PRKAR2A | 26 | 23.2 | 199 | N |
| CD4 | 27 | 47.9 | 253 | N |
| NGF | 28 | 39.5 | 201 | N |
| CRCT1 | 29 | 683.5 | 9 | N |
| IL1B | 30 | 36.7 | 269 | N |
| IFNG | 31 | 41.3 | 297 | N |
| CDH1 | 32 | 43.6 | 223 | N |
| VEGFA | 33 | 44.5 | 287 | N |
| NOD2 | 34 | 1706.6 | 47 | Y |
| BCL2 | 35 | 38.7 | 306 | N |
| PIK3R1 | 36 | 55.5 | 263 | N |
| IL23R | 37 | 1185.3 | 61 | Y |

| | | | | |
|---|---|---|---|---|
| FN1 | 38 | 24.3 | 243 | N |
| PPARG | 39 | 29.1 | 253 | N |
| RAC2 | 40 | 34.0 | 144 | N |
| ATG16L1 | 41 | 2690.1 | 20 | Y |
| PTEN | 42 | 55.5 | 198 | N |
| MAPK1 | 43 | 35.2 | 246 | N |
| CDKN2A | 44 | 37.2 | 176 | N |
| NKX2-3 | 45 | 4774.5 | 12 | Y |
| ST6GALNAC1 | 46 | 48.4 | 24 | N |
| ABCC11 | 47 | 135.3 | 51 | N |
| FURIN | 48 | 24.0 | 75 | N |
| TBP | 49 | 44.2 | 195 | N |
| DAG1 | 50 | 111.4 | 64 | N |
| GNAL | 51 | 44.0 | 298 | N |
| GRIP1 | 52 | 92.7 | 46 | N |
| GCNT1 | 53 | 41.5 | 25 | N |
| JAK2 | 54 | 104.1 | 231 | Y |
| PARK2 | 55 | 50.7 | 131 | N |
| CCND1 | 56 | 46.7 | 315 | N |
| IGF1 | 57 | 56.0 | 250 | N |
| GSK3B | 58 | 47.6 | 152 | N |
| B3GNT6 | 59 | 51.2 | 20 | N |
| GNAT3 | 60 | 124.6 | 37 | N |
| C1GALT1 | 61 | 46.5 | 21 | N |
| ACTN2 | 62 | 66.6 | 138 | N |
| VIM | 63 | 91.9 | 190 | N |
| MTF1 | 64 | 731.9 | 19 | N |
| BMP4 | 65 | 54.7 | 159 | N |
| RELA | 66 | 87.4 | 244 | N |
| MAPK8 | 67 | 55.4 | 269 | N |
| PTS | 68 | 45.6 | 46 | N |
| NOTCH1 | 69 | 52.6 | 183 | N |
| RB1 | 70 | 83.8 | 170 | N |
| RHOQ | 71 | 58.9 | 100 | N |
| RNF31 | 72 | 127.6 | 142 | N |
| PIK3CA | 73 | 84.6 | 241 | N |
| STAT1 | 74 | 100.6 | 218 | N |
| PRKACG | 75 | 68.4 | 96 | N |
| CREBBP | 76 | 153.1 | 172 | N |
| SNAP25 | 77 | 68.3 | 64 | N |

| | | | | |
|---|---|---|---|---|
| IL8 | 78 | 78.5 | 267 | N |
| ACTN4 | 79 | 137.7 | 97 | N |
| IL4 | 80 | 94.3 | 218 | N |
| TNFSF15 | 81 | 4351.7 | 16 | Y |
| CBL | 82 | 124.6 | 180 | N |
| HNF4A | 83 | 85.9 | 178 | N |
| PCNA | 84 | 70.2 | 224 | N |
| PPP2R1A | 85 | 123.6 | 124 | N |
| MGAT1 | 86 | 134.8 | 14 | N |
| CD44 | 87 | 74.9 | 217 | N |
| SLC7A13 | 88 | 3495.6 | 2 | N |
| PIK3R2 | 89 | 146.7 | 175 | N |
| ACTG1 | 90 | 200.1 | 92 | N |
| PRKCA | 91 | 70.8 | 202 | N |
| SCAPER | 92 | 5318.3 | 2 | N |
| MAPK14 | 93 | 92.5 | 193 | N |
| POMC | 94 | 135.7 | 256 | N |
| ARF6 | 95 | 101.0 | 95 | N |
| STX1A | 96 | 88.3 | 63 | N |
| PPP1CA | 97 | 115.5 | 67 | N |
| ARF1 | 98 | 105.0 | 58 | N |
| ABL1 | 99 | 93.3 | 151 | N |

**Table S1**, [a]rank_N0 is gene rank by posterior scores in networks overlaid by GWAS data (T=1 for propagaion). [b]rank_random is the average gene rank by posterior scores in networks overlaid by random prior information. [c]degree is the degree of gene in the network, [d]validate denotes whether the gene is reported (Y-Yes, N-No) as association in other Crohn's Disease studies according to GWAS catalog, www.pnas.org/cgi/doi/10.1073/pnas.0903103106

| name | $^{a}$rank_P | $^{b}$pval | $^{c}$rank_N0 | $^{d}$rank_Ni | $^{e}$validate | $^{f}$PSP |
|---|---|---|---|---|---|---|
| NOD2 | 0 | 1.76e-15 | 34 | 1432.8 | Y | 0.0000 |
| ATG16L1 | 1 | 1.08e-13 | 41 | 2311.6 | Y | 0.0000 |
| IL23R | 2 | 2.91e-13 | 37 | 1258.1 | Y | 0.0000 |
| C1orf141 | 3 | 1.15e-09 | 1853 | 5869.8 | Y | 0.0017 |
| CYLD | 4 | 4.49e-09 | 620 | 4763.9 | Y | 0.0000 |
| NKX2-3 | 5 | 1.61e-08 | 45 | 4156.3 | Y | 0.0000 |
| PTPN2 | 6 | 4.01e-07 | 234 | 3668.3 | Y | 0.0000 |
| SLC22A5 | 7 | 4.74e-07 | 224 | 3987.2 | Y | 0.0000 |
| BSN | 8 | 6.44e-07 | 351 | 1259.6 | Y | 0.0031 |
| APEH | 9 | 6.44e-07 | 2816 | 6895.4 | N | 0.0010 |
| CDKAL1 | 10 | 4.86e-06 | 768 | 2806.9 | Y | 0.0000 |
| STAT3 | 12 | 1.12e-05 | 15 | 44.3 | Y | 0.0000 |
| DAG1 | 13 | 1.27e-05 | 50 | 128.8 | N | 0.0041 |
| AMT | 15 | 1.32e-05 | 481 | 1419.5 | N | 0.0060 |
| GCKR | 19 | 1.69e-05 | 1023 | 3427.9 | Y | 0.0005 |
| IL12RB2 | 20 | 1.89e-05 | 2483 | 5702.7 | Y | 0.0006 |
| USP4 | 26 | 2.89e-05 | 2094 | 6179.7 | N | 0.0002 |
| CREM | 30 | 3.88e-05 | 422 | 2606.5 | Y | 0.0000 |
| BSG | 31 | 4.11e-05 | 1580 | 4417.7 | N | 0.0008 |
| DGKD | 33 | 4.21e-05 | 1098 | 3136.3 | Y | 0.0005 |
| ERC2 | 39 | 6.30e-05 | 827 | 3233.7 | N | 0.0085 |
| SLC22A4 | 40 | 6.77e-05 | 268 | 3734.8 | Y | 0.0000 |
| ACSL6 | 42 | 7.24e-05 | 861 | 2682.4 | Y | 0.0098 |
| IP6K1 | 46 | 7.52e-05 | 819 | 5268.6 | N | 0.0005 |
| TNFSF15 | 48 | 7.85e-05 | 81 | 4020.5 | Y | 0.0000 |
| TNFRSF6B | 51 | 8.17e-05 | 883 | 5865.4 | N | 0.0014 |
| SP110 | 54 | 8.63e-05 | 2386 | 5565.9 | N | 0.0091 |
| POU2F1 | 56 | 1.10e-04 | 2432 | 4856.6 | N | 0.0056 |
| MAPT | 61 | 1.55e-04 | 126 | 464.5 | N | 0.0007 |
| CRHR1 | 67 | 1.89e-04 | 1174 | 6646.3 | N | 0.0014 |
| WNT3 | 69 | 1.99e-04 | 1917 | 5421.9 | N | 0.0015 |
| VWF | 70 | 2.00e-04 | 658 | 1626.3 | N | 0.0058 |
| N4BP1 | 83 | 2.39e-04 | 766 | 3780.5 | N | 0.0037 |
| MOGS | 87 | 2.67e-04 | 1882 | 6474.9 | N | 0.0014 |
| GRB2 | 89 | 2.71e-04 | 24 | 36.9 | N | 0.0076 |
| MYH3 | 90 | 2.71e-04 | 2635 | 5539.0 | N | 0.0033 |
| DLG5 | 91 | 2.80e-04 | 114 | 5814.4 | N | 0.0000 |
| USP1 | 92 | 2.84e-04 | 2462 | 5958.0 | N | 0.0005 |

| | | | | | | |
|---|---|---|---|---|---|---|
| P4HA2 | 99 | 3.26e-04 | 1028 | 6499.5 | Y | 0.0001 |
| ENSP00000332488 | 101 | 3.49e-04 | 2526 | 7770.8 | N | 0.0026 |
| CD48 | 119 | 4.92e-04 | 1978 | 4466.6 | N | 0.0028 |
| PCDH15 | 128 | 5.97e-04 | 270 | 727.4 | N | 0.0067 |
| IL18RAP | 144 | 7.42e-04 | 382 | 3619.9 | Y | 0.0000 |
| PTGFR | 163 | 8.46e-04 | 1593 | 2982.6 | N | 0.0094 |
| NEDD4L | 173 | 9.08e-04 | 433 | 1213.3 | N | 0.0015 |
| GRAP2 | 175 | 9.24e-04 | 2559 | 4973.3 | N | 0.0042 |
| STX2 | 176 | 9.30e-04 | 548 | 1428.8 | N | 0.0059 |
| ADCY3 | 182 | 9.62e-04 | 403 | 895.3 | N | 0.0056 |
| IRF1 | 188 | 1.00e-03 | 781 | 2086.7 | Y | 0.0031 |
| CAMK2G | 199 | 1.10e-03 | 763 | 2074.0 | N | 0.0037 |
| IL12B | 214 | 1.28e-03 | 334 | 1887.7 | Y | 0.0000 |
| CREBBP | 227 | 1.39e-03 | 76 | 152.6 | N | 0.0016 |
| STX16 | 236 | 1.44e-03 | 1214 | 3354.3 | N | 0.0033 |
| UBE2Q1 | 256 | 1.57e-03 | 1515 | 7958.1 | N | 0.0015 |
| UTS2 | 257 | 1.57e-03 | 1818 | 4044.9 | N | 0.0022 |
| HERC2 | 259 | 1.59e-03 | 144 | 839.9 | N | 0.0004 |
| INPP5E | 261 | 1.64e-03 | 1297 | 3595.8 | N | 0.0058 |
| PTGER4 | 263 | 1.64e-03 | 749 | 2115.6 | Y | 0.0021 |
| CAPN10 | 288 | 1.91e-03 | 859 | 5083.2 | N | 0.0036 |
| DEFA5 | 293 | 1.94e-03 | 1312 | 6235.9 | N | 0.0001 |
| CDC37 | 298 | 1.97e-03 | 2517 | 5162.5 | N | 0.0070 |
| FZD6 | 303 | 2.01e-03 | 423 | 1067.2 | N | 0.0084 |
| CCNY | 305 | 2.01e-03 | 239 | 3945.7 | N | 0.0000 |
| DCTN1 | 330 | 2.19e-03 | 119 | 320.2 | N | 0.0033 |
| SLC8A1 | 331 | 2.23e-03 | 806 | 2927.4 | N | 0.0053 |
| C1orf43 | 337 | 2.25e-03 | 840 | 7057.2 | N | 0.0005 |
| POMT2 | 371 | 2.45e-03 | 614 | 2444.7 | N | 0.0075 |
| KAT2A | 373 | 2.46e-03 | 733 | 1800.2 | N | 0.0044 |
| RRAS | 408 | 2.78e-03 | 1181 | 2943.6 | N | 0.0060 |
| IL18R1 | 417 | 2.81e-03 | 1109 | 3342.2 | Y | 0.0028 |
| PIM1 | 468 | 3.34e-03 | 1123 | 3365.2 | N | 0.0011 |
| AHCYL1 | 475 | 3.42e-03 | 1801 | 4357.3 | N | 0.0093 |
| FOS | 477 | 3.43e-03 | 20 | 31.5 | N | 0.0036 |
| ASAH1 | 483 | 3.47e-03 | 1151 | 2913.4 | N | 0.0103 |
| KIAA0564 | 495 | 3.67e-03 | 805 | 2226.1 | N | 0.0098 |
| PRMT5 | 504 | 3.82e-03 | 413 | 1171.4 | N | 0.0062 |
| ST6GAL2 | 527 | 4.05e-03 | 295 | 4923.5 | N | 0.0006 |
| CHDH | 605 | 5.01e-03 | 2631 | 5462.3 | N | 0.0039 |

| | | | | | | |
|---|---|---|---|---|---|---|
| IL12RB1 | 627 | 5.23e-03 | 1432 | 3865.7 | N | 0.0002 |
| MAP3K7 | 648 | 5.42e-03 | 242 | 685.7 | N | 0.0015 |
| ZFAND6 | 673 | 5.75e-03 | 2113 | 6879.9 | N | 0.0011 |
| CD3G | 744 | 6.65e-03 | 2197 | 4901.5 | N | 0.0080 |
| CD3D | 745 | 6.65e-03 | 1045 | 3601.1 | N | 0.0006 |
| LAMA5 | 780 | 7.13e-03 | 1412 | 3080.9 | N | 0.0042 |
| SEC13 | 817 | 7.56e-03 | 399 | 921.8 | N | 0.0028 |
| RORC | 885 | 8.41e-03 | 740 | 1789.7 | N | 0.0082 |
| TRAF1 | 975 | 9.45e-03 | 380 | 1357.1 | N | 0.0001 |
| S100B | 988 | 9.59e-03 | 323 | 1093.3 | N | 0.0042 |
| ELAVL4 | 1054 | 1.09e-02 | 191 | 804.9 | N | 0.0091 |
| TWSG1 | 1057 | 1.09e-02 | 757 | 2290.9 | N | 0.0036 |
| IL15 | 1184 | 1.28e-02 | 804 | 1863.1 | N | 0.0048 |
| ABCC11 | 1232 | 1.35e-02 | 47 | 178.0 | N | 0.0002 |
| WBP4 | 1339 | 1.52e-02 | 215 | 856.7 | N | 0.0069 |
| CLCN1 | 1356 | 5.59e-03 | 975 | 3401.0 | N | 0.0054 |
| JAK1 | 1505 | 1.79e-02 | 145 | 332.3 | N | 0.0020 |
| PIP5K1A | 1589 | 1.91e-02 | 2701 | 5339.7 | N | 0.0051 |
| CTDSP2 | 1605 | 1.94e-02 | 1064 | 3294.8 | N | 0.0023 |
| SEC23B | 1608 | 1.95e-02 | 2353 | 5739.1 | N | 0.0028 |
| PHYH | 1650 | 2.01e-02 | 349 | 1540.8 | N | 0.0058 |
| PPIP5K1 | 1658 | 2.02e-02 | 472 | 3820.2 | N | 0.0000 |
| CD4 | 1731 | 2.15e-02 | 27 | 52.6 | N | 0.0022 |
| TNFRSF8 | 1766 | 2.21e-02 | 1190 | 2540.9 | N | 0.0078 |
| OSM | 1862 | 2.38e-02 | 1487 | 3085.3 | N | 0.0090 |
| CX3CR1 | 1919 | 2.48e-02 | 1459 | 3318.6 | N | 0.0068 |
| IL1R1 | 1951 | 2.55e-02 | 458 | 1599.0 | N | 0.0001 |
| SCAPER | 2034 | 2.70e-02 | 92 | 4137.7 | N | 0.0006 |
| C20orf54 | 2043 | 2.72e-02 | 1781 | 6844.2 | N | 0.0076 |
| UBFD1 | 2116 | 2.91e-02 | 330 | 1958.7 | N | 0.0073 |
| GPT | 2163 | 3.02e-02 | 2251 | 4594.1 | N | 0.0066 |
| IPPK | 2165 | 3.03e-02 | 905 | 4736.9 | N | 0.0006 |
| LIMCH1 | 2242 | 3.17e-02 | 170 | 3303.0 | N | 0.0101 |
| SOCS4 | 2330 | 3.36e-02 | 1021 | 2636.7 | N | 0.0035 |
| NCKAP1 | 2361 | 3.43e-02 | 865 | 3057.7 | N | 0.0069 |
| CD3E | 2621 | 4.04e-02 | 1654 | 3943.6 | N | 0.0051 |
| FKBP4 | 2704 | 4.33e-02 | 2629 | 6167.0 | N | 0.0020 |
| TMC8 | 2869 | 4.74e-02 | 1812 | 7839.2 | N | 0.0043 |
| TMC6 | 2870 | 4.74e-02 | 1811 | 7700.6 | N | 0.0045 |
| FAM92B | 2880 | 4.75e-02 | 102 | 5654.4 | N | 0.0001 |

| | | | | | | |
|---|---|---|---|---|---|---|
| SSU72 | 2885 | 4.78e-02 | 416 | 3835.6 | N | 0.0000 |
| SEC24D | 2933 | 4.88e-02 | 2360 | 5425.9 | N | 0.0085 |
| IP6K3 | 3052 | 5.21e-02 | 1042 | 6119.4 | N | 0.0001 |
| JAK2 | 3063 | 5.23e-02 | 54 | 100.2 | Y | 0.0025 |
| IL5 | 3137 | 5.43e-02 | 440 | 1009.2 | N | 0.0101 |
| UBAC1 | 3232 | 5.64e-02 | 1081 | 5968.7 | N | 0.0027 |
| SEC23A | 3285 | 5.80e-02 | 1500 | 4870.5 | N | 0.0031 |
| RHOC | 3352 | 5.97e-02 | 1463 | 4109.1 | N | 0.0089 |
| PTPN22 | 3390 | 6.09e-02 | 651 | 2025.7 | Y | 0.0015 |
| ESYT3 | 3425 | 6.20e-02 | 2424 | 6008.4 | N | 0.0091 |
| GABARAPL2 | 3580 | 6.62e-02 | 546 | 2401.3 | N | 0.0064 |
| HIST1H4A | 3616 | 6.71e-02 | 1860 | 6167.5 | N | 0.0008 |
| TMEM39A | 3650 | 6.81e-02 | 530 | 6071.9 | N | 0.0016 |
| IL13 | 3734 | 7.07e-02 | 932 | 2662.1 | N | 0.0022 |
| PPAP2A | 3741 | 7.09e-02 | 193 | 543.5 | N | 0.0044 |
| C10orf107 | 3760 | 7.17e-02 | 634 | 2249.5 | N | 0.0066 |
| FECH | 3825 | 7.37e-02 | 1933 | 4514.8 | N | 0.0071 |
| ACTG1 | 4576 | 1.01e-01 | 90 | 200.7 | N | 0.0084 |
| IL23A | 4657 | 1.04e-01 | 489 | 1467.1 | N | 0.0012 |
| CXCR4 | 4821 | 1.11e-01 | 252 | 598.3 | N | 0.0088 |
| SLC7A13 | 5476 | 1.41e-01 | 88 | 3944.4 | N | 0.0005 |
| RIMS2 | 5769 | 1.56e-01 | 345 | 1720.0 | N | 0.0007 |
| KPNA7 | 5815 | 7.68e-02 | 2007 | 7231.5 | N | 0.0048 |
| CRCT1 | 6411 | 1.94e-01 | 29 | 1282.8 | N | 0.0003 |
| PMM2 | 6910 | 2.27e-01 | 496 | 1918.6 | N | 0.0060 |
| PPIP5K2 | 8248 | 3.29e-01 | 1611 | 5902.5 | N | 0.0005 |
| C1orf64 | 8316 | 3.35e-01 | 637 | 7746.2 | N | 0.0033 |
| FASLG | 8483 | 3.54e-01 | 227 | 711.3 | Y | 0.0007 |
| TRAPPC1 | 9276 | 4.48e-01 | 1972 | 6522.3 | N | 0.0060 |
| GKN2 | 10028 | 5.55e-01 | 589 | 4442.7 | N | 0.0033 |
| MTERF | 10454 | 1.61e-01 | 2818 | 8682.0 | N | 0.0075 |
| IGHJ6 | 10958 | 7.78e-01 | 324 | 6174.5 | N | 0.0060 |

**Table S2**, [a]rank_P is rank of genes based on gene level $p$ values from GWAS, [b]pval is $p$ values from GWAS, [c]rank_N0 is rank of posterior scores in network overlaid by GWAS prior, [d]rank_Ni is average gene rank by posterior scores in networks overlaid by random prior information. [e]validate denotes whether the gene is reported (Y-Yes, N-No) as association in other Crohn's Disease studies according to GWAS catalog, www.pnas.org/cgi/doi/10.1073/pnas.0903103106. [f] is the $p$ value of posterior score, based on 10000 permutations, calculated as the proportion of occurrence of larger posterior scores in the permutation.

| IL12_pathway | rank_GWAS | [a]rank_N0 | [b]rank_random |
|---|---|---|---|
| JUN | 2916 | 16 | 18.2 |
| MAP2K6 | 2025 | 970 | 718.2 |
| IL12A | 9016 | 3527 | 4461.9 |
| IL12B | 214 | 334 | 1887.7 |
| CD3E | 2621 | 1654 | 3943.6 |
| CD3G | 744 | 2197 | 4901.5 |
| MAPK8 | 9032 | 67 | 56.8 |
| ETV5 | 6467 | 1994 | 1884.1 |
| IFNG | 9837 | 31 | 44.9 |
| STAT4 | 5617 | 1007 | 1323.9 |
| IL12RB1 | 627 | 1432 | 3865.7 |
| MAPK14 | 3010 | 93 | 93.0 |
| CD247 | 2277 | 1016 | 1756.3 |
| IL12RB2 | 20 | 2483 | 5702.7 |
| IL18 | 5164 | 293 | 612.7 |
| TYK2 | 9622 | 499 | 781.9 |
| JAK2 | 3063 | 54 | 100.2 |
| IL18R1 | 417 | 1109 | 3342.2 |
| CD3D | 745 | 1045 | 3601.1 |

**Table S3** Ranks of IL12 pathway genes in GWAS and network propagation ($T = 1$), GWAS rank of gene is based on the most significant SNPs. [a]rank_N0 is gene rank by posterior scores in network of GWAS prior. [b]rank_random is the average gene rank by posterior scores in networks of random prior. 15 out of 19 genes have a rank increase in the network of GWAS prior.
